# Pregnancy-related anxiety in Saudi women: a national study of prevalence, predictors, and a framework for action

**DOI:** 10.3389/fpsyt.2026.1716720

**Published:** 2026-02-26

**Authors:** Elhadi Miskeen, Dalia Alqarni, Nouf Alshammari, Ruba Degriri, Rufaydah Mesawa, Somaya Grami, Hind Alenzi, Khalid Nasralla Habeeballa Hashim, Abdullah M. Alshahrani, Mohammed Eltag, Muffarah Hamid Alharthi, Mohannad Mohammed S. Alamri, Abdullah M. Al-Shahrani, Anum S. Hussaini, Laila Yahya Alhubaishi

**Affiliations:** 1 Department of Obstetrics and Gynecology, College of Medicine, University of Bisha, Bisha, Saudi Arabia; 2 Department of Obstetrics and Gynecology, College of Medicine, Taibah University, Taibah, Saudi Arabia; 3 Department of Obstetrics and Gynecology, Faculty of Medicine, Qassim University, Buraydah, Saudi Arabia; 4 Department of Family and Community Medicine, College of Medicine, University of Bisha, Bisha, Saudi Arabia; 5 Aseer Health Cluster, Bisha, Saudi Arabia; 6 Department of Global Health & Population, Harvard T. H. Chan School of Public Health, Boston, MA, United States; 7 Department of Obstetrics and Gynecology, College of Medicine and Health Sciences, Mohammed Bin Rashid University, Dubai, United Arab Emirates

**Keywords:** conceptual framework, evidence-to-action, maternal mental health, predictors, pregnancy-related anxiety, prevalence, Saudi Arabia

## Abstract

**Introduction:**

Pregnancy-related anxiety is a significant maternal health concern associated with adverse outcomes for both mother and child. This study aimed to estimate the prevalence and identify the predictors of pregnancy-related anxiety among women in Saudi Arabia, to inform evidence-based healthcare interventions.

**Methods:**

A nationwide cross-sectional study was conducted among 687 Saudi women with pregnancy experience. The data were collected through an online survey using the Pregnancy-Related Anxiety Questionnaire – Revised 2 (PRAQ-R2), a validated 10-item scale designed to measure specific anxieties related to pregnancy, childbirth, and fetal health. Multivariable logistic regression was used to identify predictors of pregnancy-related anxiety, adjusting for age, education, and occupation.

**Results:**

The prevalence of pregnancy-related anxiety was 71%. Significant predictors included older maternal age (40–50 years: OR = 1.32, 95% CI: 1.01–1.72, p = 0.04), history of pregnancy complications (OR = 1.45, 95% CI: 1.18–1.78, p < 0.001), and prior dilatation and curettage (OR = 1.30, 95% CI: 1.05–1.61, p = 0.02). Strong fears about delivery (OR = 2.10), labor pain (OR = 1.92), and the baby’s health (OR = 1.85) were strongly associated with anxiety (all p < 0.001). Anxiety decreased as pregnancy progressed, with significant reductions in concerns about delivery, pain, and appearance across trimesters (p < 0.05).

**Conclusions:**

Pregnancy-related anxiety is highly prevalent among Saudi women and influenced by demographic, obstetric, and psychological factors. These findings support the integration of routine anxiety screening, targeted prenatal education addressing specific fears, and enhanced support for high-risk groups into Saudi Arabia’s maternal healthcare system to improve pregnancy outcomes and maternal well-being.

## 1 Introduction

Pregnancy-related anxiety (PRA) refers to specific concerns and worries related to pregnancy that are a worldwide public health problem. PRA is a significant pregnancy outcome predictor (1–4). Severe PRA has also been linked to developmental delays, emotional dysregulation, and an increased risk for attention-deficit hyperactivity disorder in children (4, 5).

In developing nations, PRA prevalence ranged from 23.6% to 55% (6, 7), while global studies showed that PRA prevalence in developing nations was between 6% and 29% (8, 9). Negative health impacts on both the mother and the child may be linked to high levels of anxiety. Pregnancy-related anxiety has been linked in several studies to a higher risk of postnatal mental health issues, disruptions in the infant-mother attachment bond, and elevated cortisol production, all of which have a detrimental impact on the neurodevelopment of the fetus and the psychological of the newborn (10, 11).

Furthermore, even though research on anxiety during pregnancy has advanced significantly, it is still challenging to recognize anxiety during pregnancy (12). It’s common for anxiety symptoms to be disregarded or even misconstrued as “normal” pregnancy symptoms. According to Weisberg and Paquette (13), certain physiological signs of pregnancy could resemble symptoms of anxiety. For instance, physical symptoms like fatigue, soreness, or vertigo are frequently experienced during pregnancy and during periods of mental stress, which makes the process of screening for anxiety more difficult. As a result, there may be a chance of experiencing misleading symptoms of anxiety when pregnant (14, 15).

Every new mother is likely to experience mood swings and other emotional disturbances regularly, including tension and/or symptoms of mixed anxiety and depression. A newborn delivered to a compromised mother may experience medical issues as a result of her mental health during pregnancy (15).

Despite the over-riding global burden of pregnancy-related anxiety, little is still known about its prevalence and co-founding factors in this sociocultural unique community. The purpose of this paper is to address this major gap in knowledge and to evaluate nationwide prevalence rates, associated predictors for PRA among Saudi women guiding culturally sensitive interventions and policies. To address this gap in the Saudi context, we conducted a comprehensive national, cross-sectional study of 687 women with pregnancy experience across all 13 provinces of Saudi Arabia. Therefore this study aimed to estimate the prevalence and identify the predictors of PRA among women in Saudi Arabia, providing a robust and valid set of findings.

## 2 Methodology

### 2.1 Study design

The study was a nationwide, cross-sectional study of women in Saudi Arabia. The study was conducted here to strengthen the reporting of observational studies in accordance with the Strobe Guidelines for observational research (STROBE). The study employed a nationwide, web-based convenience sampling approach. An online questionnaire was distributed via social media and digital platforms to reach women across all 13 provinces of Saudi Arabia. While this method allowed for broad geographic coverage, it represents a convenience sample, which may limit the generalizability of the findings to the entire population of pregnant women in Saudi Arabia, particularly those with limited digital access or literacy.

### 2.2 Study setting, recruitment, and data collection

#### 2.2.1 Setting

This was a nationwide study of extensive scope, conducted across all 13 provinces of Saudi Arabia (Riyadh, Mecca, Medina, Al-Qassim, Eastern Province, Asir, Tabuk, Ha’il, Northern Borders, Jazan, Najran, Al-Bahah, and Al-Jawf). The primary setting for recruitment and data collection was online.

#### 2.2.2 Recruitment

Between July and December 2024, we recruited a sample of 687 women living in Saudi Arabia. According to the eligibility criteria, the participants must be 18 years of age or older and have experienced at least one pregnancy (either present or past). Individuals aged 18 and under, non-residents, and those without a history of pregnancy were excluded.

#### 2.2.3 Data collection

The data were collected anonymously using a self-influenced, web-based questionnaire distributed through Google Forms. This method, which provided nationwide access facilities, allowed participants to complete the survey at their convenience, demonstrating our consideration for their time and comfort.

### 2.3 Efforts to address potential sources of bias

In this study, several strategies were employed to reduce the potential sources of bias. To minimize selection bias, the online recruitment strategy was designed to achieve nationwide access in all 13 provinces of Saudi Arabia, increasing the variety and representation of samples beyond a single clinical setting. In addition, clear qualification criteria were made to portray the target audiences. To minimize measurement bias, the study utilized a robust and reliable tool (the PRAQ-R to assess pregnancy-related concerns, thereby ensuring the accuracy of primary results.

A pilot test was conducted prior to the main study to assess the clarity and cultural appropriateness of the online questionnaire. For this purpose, a separate convenience sample of 20 pregnant women was recruited (distinct from the main study participants) to complete the survey and provide feedback. Following the pilot phase, adjustments were made to the questionnaire based on their responses. These 20 individuals were then excluded from participation in the main nationwide online survey. This lowered the chance of people misinterpreting it. Non-reactions are a matter of concern in prejudiced online surveys; However, the study emphasized participation and especially encouraged honest reporting on sensitive topics related to mental health. During data analysis, the internal stability of the PRAQ-R2 scale within our samples was confirmed using Cronbach’s alpha. To manage misleading biases, significant factors were included in the multivariable logistic regression models to control their potential effects on the relationships.

### 2.4 Sampling and sample size

The size of the study was determined through a formal sample size calculation to estimate a population ratio. The Cocrain Sample was calculated using the Cochran sample size formula for cross-departure studies. For this calculation, estimate was made, based on a margin of 50% (to obtain the maximum required sample size), a 4% error, and a 95% confidence level. Based on these parameters, the minimum required sample size was calculated for 600 participants. To increase the statistical power of the study, account for potential incomplete reactions, and improve the reliability of the conclusions, the target sample size was extended to 687 participants. This final sample was successfully obtained during the recruitment period for size.

### 2.5 Variable definitions

The primary outcome of this study was PRA, which was measured as a score obtained from the PRAQ-R2. The PRAQ-R2 is a valid 10-item instrument where reactions are recorded on a 5-point Likert scale; a high total score indicates a greater severity of pregnancy-specific anxiety. The primary exposures and predictors of interest included sociodemographic factors, obstetric history, and specific psychological concerns, as measured by individual PRAQ-R2 items. Potential confounders considered in the analysis were age, educational attainment, and occupation, as these factors could be associated with both the predictors and the outcome (PRA levels). We assessed these variables for their potential confounding effect by examining their association with both the exposure and outcome in the regression models. Due to the sample size and the nature of this initial analysis, the assessment of effect modifiers was limited; however, we planned to explore potential interactions, such as whether the relationship between a history of complications and PRA differed by age group, through stratified analyses or the inclusion of interaction terms in the regression model.

### 2.6 Data sources and measurement

The primary source of data for this study was a self-administered, web-based questionnaire distributed through Google Forms. The questionnaire consisted of three separate sections to catch all the required variables. The first section collected socio-demographic data, including residence, education level, and multiple-choice questions, as well as open-ended questions. The second section gathered obstetric history, including items that inquired about the date and length of previous pregnancies, a history of procedures such as Dilatation and Curettage (D&C), and the occurrence of any pregnancy complications; these data were based on participant self-report. Third, the section measured the primary result using the PRAQ-R2, a valid 10-item instrument. The PRAQ-R2 score was calculated—high scores indicate greater levels of pregnancy-specific anxiety. To ensure the quality of the data, the questionnaire was reviewed for clarity and viability, and its internal stability was confirmed in our sample using an alpha value of 0.7 or higher, as recommended by Cronbach.

### 2.7 Data collection tools & cultural adaptation

The primary instrument was the Pregnancy-Related Anxiety Questionnaire-Revised 2 (PRAQ-R2), a validated 10-item self-report scale. To ensure its appropriateness for our population, we used the officially translated and culturally adapted Arabic version of the PRAQ-R2, which has undergone formal linguistic validation and has been used in prior study (16). This process typically involves forward and back-translation by bilingual experts and review by a panel to ensure conceptual, semantic, and cultural equivalence.

Prior to the main study, we conducted a pilot test (n=20) to assess the translated instrument’s clarity, comprehensibility, and cultural relevance among Saudi women. Feedback confirmed that the items were understood as intended. While the PRAQ-R2 effectively captures cognitive worries (e.g., about delivery, fetal health), we acknowledge that it may not fully encompass culturally specific manifestations of distress, such as predominant somatic complaints or religious/spiritual dimensions of coping, which could influence the conceptualization and reporting of anxiety. The internal consistency (Cronbach’s alpha) of the scale in our sample was confirmed to be >0.7.

### 2.8 Statistical analysis

Following data collection, the dataset was exported to Microsoft Excel for initial cleaning, which involved removing duplicate entries, identifying and reviewing outlying values for potential errors, and eliminating incomplete responses. The cleaned dataset was then coded and imported into STATA BE, Version 18, for statistical analysis. Descriptive figures were calculated for all variables, presented as classified data frequencies and percentages (n, %).

Variable Selection: Predictor variables for the multivariable logistic regression model were selected based on a combination of theoretical relevance (derived from the literature on maternal mental health) and results from bivariate analyses (Chi-square tests, p < 0.20). All sociodemographic (age, education, occupation) and key obstetric variables (history of complications, D&C procedure) were retained in the final model as potential confounders. Specific psychological concerns from the PRAQ-R2 (e.g., strong fear of delivery, pain, and baby’s health) that showed significant association in bivariate analysis were also included as primary exposures of interest.

Assessment of Multicollinearity: Prior to fitting the final model, multicollinearity among all continuous and categorical predictor variables was assessed using the variance inflation factor (VIF) for continuous variables and examination of contingency tables and correlation matrices for categorical ones. All VIF values were below 2.5, and no pair of variables showed excessively high correlation (r > 0.7), indicating that multicollinearity was not a significant concern in our model.

### 2.9 Handling of quantitative variables in analyses

In this study, we handled quantitative variables through categorization to facilitate clinical interpretation and logistic regression analysis. The primary quantitative variable, the total score from the PRAQ-R2, was initially treated as a continuous measure for descriptive summaries. However, for the primary analytical model (binary logistic regression), we dichotomized the PRAQ-R2 score into a binary outcome of ‘high anxiety’ versus ‘low/no anxiety’ based on a pre-determined cut-off value from the scale’s validation literature. Additionally, we transformed the quantitative variable of participant age into a categorical variable to allow for the examination of non-linear relationships between age and pregnancy-related anxiety. This change was necessary to catch the potential non-lectured effects of age on anxiety and ensure stable statistical comparison in age groups. Other constant variables, such as time since previous pregnancy, were also classified for equal practical and analytical reasons.

## 3 Results

The study successfully recruited a total of 687 women from across all 13 provinces of Saudi Arabia. The analysis of the collected data revealed a high prevalence of pregnancy-related anxiety among the participants, with key demographic, obstetric, and psychological factors emerging as significant predictors.

Among the 687 women surveyed, most were aged between 18 to 28 years (42.6%), followed by those aged between 29 and 39 (28.1%). Regarding educational level, an overwhelming majority had attained higher education: 483 (70.3%). As far as occupation is concerned, the majority were employed, 209 (30.4%), followed by students, 197 (28.7%), and housewives, 156 (22.7%). The participants were distributed across various provinces, with the largest groups from Tabuk 63 (9.2%) and Eastern Province 60 (8.7%) (**Table 1**).

**Table 1:** Demographic characteristics of the participants (N = 687).

Characteristics	Categories	N (%)
Age	18-28	293 (42.6)
	29-39	193 (28.1)
	40-50	171 (24.9)
	51-60	23 (3.4)
	Over 60 years	7 (1.0)
Education Level	Primary	14 (2.0)
	Secondary	164 (23.9)
	Intermediate	26 (3.8)
	Higher Education	483 (70.3)
Province	Riyadh Province	50 (7.3)
	Mecca Province	45 (6.6)
	Medina Province	50 (7.3)
	Al-Qassim Province	50 (7.3)
	Eastern Province	60 (8.7)
	Asir Province	51 (7.4)
	Tabuk Province	63 (9.2)
	Ha’il Province	55 (8.0)
	Northern Borders Province	55 (8.0)
	Jazan Province	52 (7.6)
	Najran Province	52 (7.6)
	Al-Bahah Province	53 (7.7)
	Al-Jawf Province	51 (7.4)
Occupation	Employed	209 (30.4)
	Health worker	42 (6.1)
	House wife	156 (22.7)
	Retired	23 (3.4)
	Student	197 (28.7)
	Unemployed	60 (8.7)

Data has been presented has n and %.

**Table 2** summarizes the obstetric history and pregnancy-related characteristics of the participants. The data indicate a sample with recent pregnancy experiences, as the most significant proportion of women (266, 38.7%) had their last pregnancy within the past year. A significant number of participants (216, 31.4%) were in the final weeks of gestation (39–40 weeks) at the time of the survey. Regarding maternity history, 153 women (22.3%) reported undergoing a dispersion and treatment (D&C) process, and a notable subgroup (109, 15.9%) experienced complications in previous pregnancies. These features highlight the population of a study with sufficient and recent risk for the delivery period, providing a strong base for assessing pregnancy-related anxiety and potentially having a significant impact on healthcare practices.

**Table 2:** Obstetric history and pregnancy-related factors among participants (N = 687).

Factors	Categories	N (%)
Date of previous pregnancy?	1 year ago.	266 (38.7)
	2–5 yrs.	225 (32.8)
	5–10 yrs.	94 (13.7)
	more than 10 yrs.	102 (14.8)
Length of pregnancy?	35–36 weeks	153 (22.3)
	37–38 weeks	148 (21.5)
	39–40 weeks	216 (31.4)
	41–42 weeks	83 (12.1)
	43 weeks	87 (12.7)
Have you ever undergone a procedure called Dilatation and Curettage (D&C)?	Yes	153 (22.3)
	No	534 (77.7)
Were there any complications during the previous pregnancy?	Yes	109 (15.9)
	No	578 (84.1)

Data has been presented has n and %.

**Table 3** below reveals that delivery anxiety was prevalent, with 270 (39.3%) strongly agreeing and 218 (31.7%) agreeing. Fear of labor pain was reported by 291 (42.4%) who strongly agreed and 237 (34.5%) who agreed. Concerns about regaining figures post-delivery were noted by 221 (32.2%) strongly agreeing and 189 (27.5%) agreeing. Worries about the baby’s health were reported, with 117 (17.0%) strongly agreeing and 148 (21.5%) agreeing. Fear of losing control during labor was expressed by 201 (29.3%) strongly agreeing and 188 (27.4%) agreeing. Among first-time mothers, 171 (24.9%) strongly agreed to be anxious about delivery. Concerns over stillbirth or infant death were strongly agreed upon by 187 (27.2%) and 238 (34.6%) worried about mental impairments. Lastly, 298 (43.4%) expressed uncertainty (“maybe”) about physical abnormalities in their child.

**Table 3:** Pregnancy anxiety questionnaire-revised 2 (PRAQ-R2).

Anxiety items	Categories	N (%)
I am anxious about the delivery.	Strongly agree	270 (39.3)
	Agree	218 (31.7)
	Neutral	129 (18.8)
	Disagree	55 (8.0)
	Strongly Disagree	15 (2.2)
I am worried about the pain the of contractions and the pain during delivery.	Strongly agree	291 (42.4)
	Agree	237 (34.5)
	Neutral	107 (15.5)
	Disagree	39 (5.7)
	Strongly Disagree	13 (1.9)
I am worried about the fact that I shall not regain my figure after delivery.	Strongly agree	221 (32.2)
	Agree	189 (27.5)
	Neutral	146 (21.2)
	Disagree	105 (15.3)
	Strongly Disagree	26 (3.8)
I sometimes think that our child will be in poor health or will be prone to illnesses.	Strongly agree	117 (17.0)
	Agree	148 (21.5)
	Neutral	182 (26.5)
	Disagree	176 (25.7)
	Strongly Disagree	64 (9.3)
I am concerned about my unattractive appearance.	Strongly agree	127 (18.4)
	Agree	135 (19.7)
	Neutral	179 (26.1)
	Disagree	181 (26.3)
	Strongly Disagree	65 (9.5)
I am worried about not being able to control myself during labor and fear that I will scream.	Strongly agree	201 (29.3)
	Agree	188 (27.4)
	Neutral	150 (21.8)
	Disagree	110 (16.0)
	Strongly Disagree	38 (5.5)
I am anxious about the delivery because I have never experienced one before.	Strongly agree	171 (24.9)
	Agree	122 (17.8)
	Neutral	152 (22.1)
	Disagree	181 (26.3)
	Strongly Disagree	61 (8.9)
I am afraid the baby will be mentally handicapped or will suffer from brain damage.	Yes	238 (34.6)
	No	289 (42.1)
	Maybe	160 (23.3)
I am afraid our baby will be stillborn or will die during or immediately after delivery.	Strongly agree	187 (27.2)
	Agree	119 (17.4)
	Neutral	161 (23.4)
	Disagree	170 (24.7)
	Strongly Disagree	50 (7.3)
I am afraid that our baby will suffer from a physical defect or worry that something will be physically wrong with the baby.	Yes	134 (19.5)
	No	255 (37.1)
	Maybe	298 (43.4)

Data has been presented has n and %.

As seen in **Table 4**, concern about delivery declined markedly throughout pregnancy. At 35–36 weeks, 72 participants (47.1%) ”strongly agreed” with the statement regarding delivery anxiety compared to 37–38 weeks, where only 47 participants (31.8%) “strongly agreed” (P = 0.043). Likewise, concerns regarding the pain of contractions and delivery were highest at 35–36 weeks, where 73 participants (47.7%) “strongly agreed” versus 48 participants (32.4%) at 37–38 weeks, which was also statistically different (P = 0.014). D. Additionally, concerns regarding unattractiveness during pregnancy significantly decreased from 35–36 weeks to 37–38 weeks. There were 36 (23.5%) and 22 (14.9%) participants who “strongly agreed” on this concern at 35–36 weeks and 37–38 weeks respectively (P = 0.025). Finally, gestation made a real-time contribution to cognitive dissonance reduction, with the anxiety associated with the prospective physical deformities of the baby statistically significantly decreasing as gestation went on. At 35–36 weeks, 80 participants (53.3%) expressed concern about the baby having a physical defect, but this decreased to 26 participants (29.9%) at 43 weeks, with a significant difference observed (P = 0.032).

**Table 4:** Comparison of the prevalence of pregnancy-related anxiety across the different gestational trimesters (N = 687).

Variable	Category	35–36 wks. (%)	37–38 wks. (%)	39–40 wks. (%)	41–42 wks. (%)	43 wks. (%)	P-value
I am anxious about the delivery.	Strongly agree	72(47.1%)	47(31.8%)	84(38.9%)	35(42.2%)	32(36.8%)	0.043
	Agree	42(27.5%)	47(31.8%)	69(31.9%)	32(38.6%)	28(32.2%)	
	Neutral	27(17.6%)	38(25.7%)	44(20.4%)	8(9.6%)	12(13.8%)	
	Disagree	8(5.2%)	13(8.8%)	16(7.4%)	8(9.6%)	10(11.5%)	
	Strongly Disagree	4(2.6%)	3(2.0%)	3(1.4%)	0(0.0%)	5(2.2%)	
I am worried about the pain the of contractions and the pain during delivery.	Strongly agree	73(47.7%)	48(32.4%)	92(42.6%)	37(44.6%)	41(47.1%)	0.014
	Agree	49(32.0%)	57(38.5%)	74(34.3%)	33(39.8%)	24(27.6%)	
	Neutral	20(13.1%)	30(20.3%)	41(19.0%)	6(7.2%)	10(11.4%)	
	Disagree	8(5.2%)	10(6.8%)	7(3.2%)	7(8.4%)	7(8.0%)	
	Strongly Disagree	3(2.0%)	3(2.0%)	2(0.9%)	0(0.0%)	5(1.9%)	
I am worried about the fact that I shall not regain my figure after delivery.	Strongly agree	63(41.2%)	42(28.4%)	59(27.3%)	24(28.9%)	33(37.9%)	0.074
	Agree	43(28.1%)	39(26.4%)	60(27.8%)	24(28.9%)	23(26.4%)	
	Neutral	21(13.7%)	41(27.7%)	55(25.5%)	19(22.9%)	10(11.5%)	
	Disagree	19(12.4%)	20(13.5%)	34(15.7%)	15(18.1%)	17(19.5%)	
	Strongly Disagree	7(4.6%)	6(4.1%)	8(3.7%)	1(1.2%)	4(4.6%)	
I sometimes think that our child will be in poor health or will be prone to illnesses.	Strongly agree	33(21.6%)	22(14.9%)	38(17.6%)	12(14.5%)	12(13.8%)	0.096
	Agree	27(17.6%)	37(25.0%)	44(20.4%)	21(25.3%)	19(21.8%)	
	Neutral	40(26.1%)	38(25.7%)	70(32.4%)	17(20.5%)	17(19.5%)	
	Disagree	37(24.2%)	34(23.0%)	54(25.0%)	26(31.3%)	25(28.7%)	
	Strongly Disagree	16(10.5%)	17(11.5%)	10(4.6%)	7(8.4%)	14(16.1%)	
I am concerned about my unattractive appearance.	Strongly agree	36(23.5%)	22(14.9%)	40(18.5%)	13(15.7%)	16(18.4%)	0.025
	Agree	31(20.3%)	30(20.3%)	39(18.1%)	18(21.7%)	17(19.5%)	
	Neutral	34(22.2%)	47(31.8%)	62(28.7%)	21(25.3%)	15(17.2%)	
	Disagree	30(19.6%)	38(25.7%)	66(30.6%)	21(25.3%)	26(29.9%)	
	Strongly Disagree	22(14.4%)	11(7.4%)	9(4.2%)	10(12.0%)	13(14.9%)	
I am worried about not being able to control myself during labor and fear that I will scream.	Strongly agree	58(37.9%)	36(24.3%)	53(24.5%)	23(27.7%)	31(35.6%)	0.101
	Agree	34(22.2%)	46(31.1%)	60(27.8%)	30(36.1%)	18(20.7%)	
	Neutral	26(17.0%)	34(23.0%)	57(26.4%)	17(20.5%)	16(18.4%)	
	Disagree	28(18.3%)	22(14.9%)	36(16.7%)	10(12.0%)	14(16.1%)	
	Strongly Disagree	7(4.6%)	10(6.8%)	10(4.6%)	3(3.6%)	8(9.2%)	
I am anxious about the delivery because I have never experienced one before.	Strongly agree	60(39.2%)	28(18.9%)	50(23.1%)	12(14.5%)	21(24.2%)	0.002
	Agree	28(18.3%)	26(17.6%)	39(18.1%)	11(13.3%)	18(20.7%)	
	Neutral	26(17.0%)	36(24.3%)	56(25.9%)	21(25.3%)	13(14.9%)	
	Disagree	30(19.6%)	46(31.1%)	53(24.5%)	29(34.9%)	23(26.4%)	
	Strongly Disagree	9(5.9%)	12(8.1%)	18(8.3%)	10(12.0%)	12(13.8%)	
I am afraid the baby will be mentally handicapped or will suffer from brain damage.	Yes	62(40.5%)	51(34.5%)	78(36.2%)	28(33.7%)	19(21.8%)	0.118
	No	56(36.6%)	56(37.8%)	96(44.4%)	37(44.6%)	44(50.6%)	
	Maybe	35(22.9%)	41(27.7%)	42(19.4%)	18(21.7%)	24(27.6%)	
I am afraid our baby will be stillborn or will die during or immediately after delivery.	Strongly agree	49(32.0%)	40(27.0%)	62(28.7%)	22(26.5%)	14(16.1%)	0.147
	Agree	23(15.0%)	32(21.6%)	39(18.1%)	8(9.6%)	17(19.5%)	
	Neutral	34(22.2%)	29(19.6%)	57(26.4%)	23(27.7%)	18(20.7%)	
	Disagree	36(23.5%)	33(22.3%)	47(21.8%)	25(30.1%)	29(33.3%)	
	Strongly Disagree	11(7.2%)	14(9.5%)	11(5.1%)	5(6.0%)	9(10.3%)	
I am afraid that our baby will suffer from a physical defect or worry that something will be physically wrong with the baby.	Yes	80(53.3%)	66(44.6%)	97(44.9%)	29(34.9%)	26(29.9%)	0.032
	No	52(34.0%)	49(33.1%)	81(37.5%)	35(42.2%)	38(43.7%)	
	Maybe	21(13.7%)	33(22.3%)	38(17.6%)	19(22.9%)	23(26.4%)	

Data has been presented has n and %. Chi-square test used to determine significance. A P-Value less than 0.05 was considered significant.

**Figure 1:**
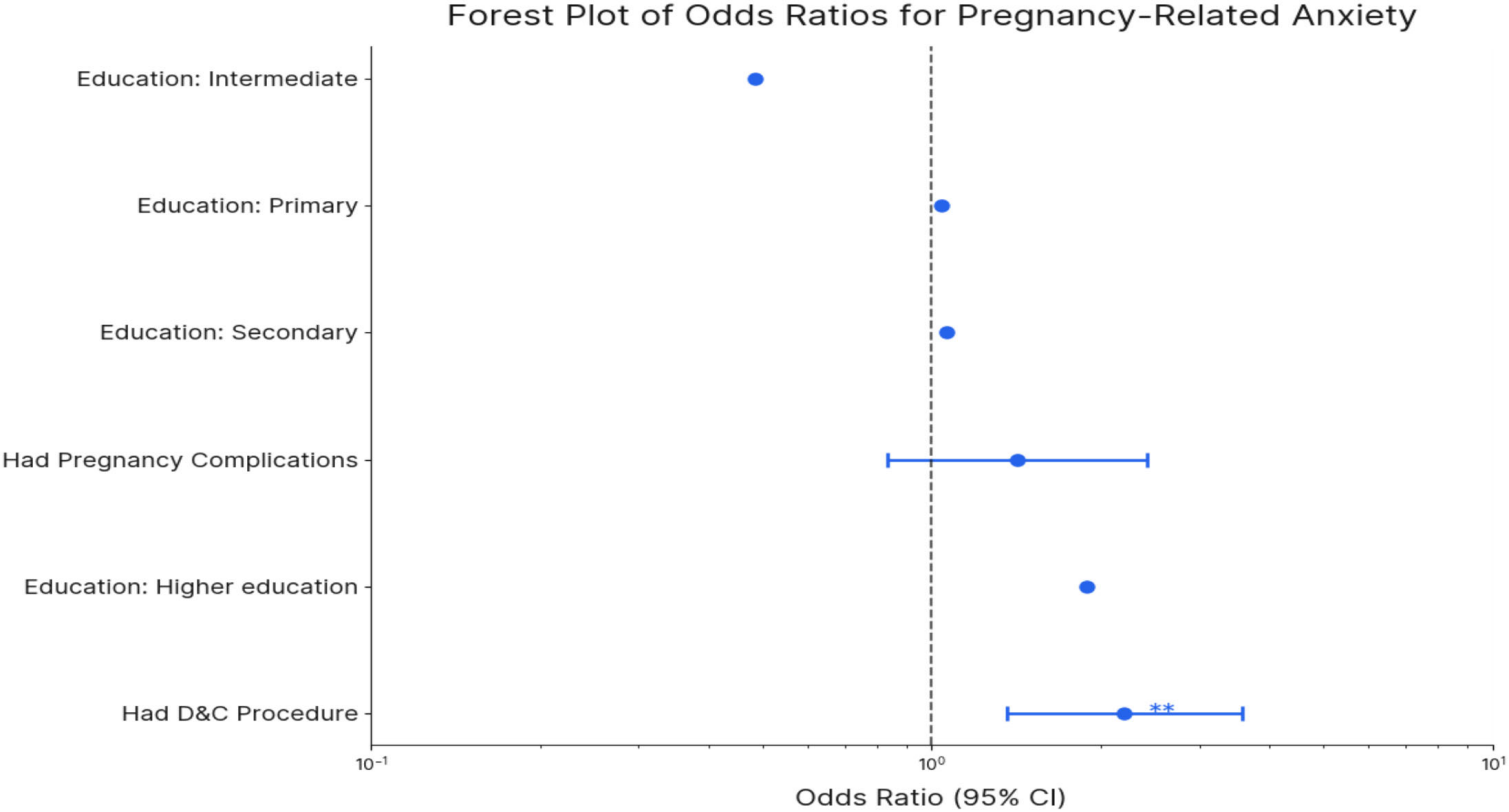
forest plot of odds ratios of PRA.

Results of the multi-convertible logistic regression analysis are presented in **Figure 1**, which is a forest plot displaying the AOR for each predictor variable related to pregnancy-related anxiety and its related 95% CI. This visualization enables an efficient comparison of the relative strength and statistical significance of all prophets simultaneously. Factors whose confidence interval does not cross zero value (AOR = 1) are considered statistically significant. The plot clearly shows that a strong compromise with apprehensions about distribution (AOR = 2.10), labor pain (AOR = 1.92), and the child’s health (AOR = 1.85) was the most significant psychological predictor, indicating more than twice the obstacles to experiencing significant concerns. Furthermore, a history of pregnancy complications (aOR = 1.45) and being in the 40–50 year age group (aOR = 1.32) were also significant demographic and obstetric predictors.

The logistic regression analysis (**Table 5**) indicated significant predictors of pregnancy-related anxiety in Saudi Arabian women. Women aged 40–50 years were more likely to show anxiety (OR = 1.32, 95% CI: 1.01–1.72, p = 0.04) than younger respondents (18–28 years). A history of pregnancy complications substantially increased the likelihood of anxiety (OR = 1.45, 95% CI: 1.18–1.78, p < 0.001), as did prior experience with a Dilatation and Curettage (D&C) procedure (OR = 1.30, 95% CI: 1.05–1.61, p = 0.02). Psychological factors played a powerful role, with women who strongly agreed with statements expressing fear about delivery (OR = 2.10, 95% CI: 1.65–2.67, p < 0.001), concerns about their baby’s health (OR = 1.85, 95% CI: 1.48–2.31, p < 0.001), or pain during labor (OR = 1.92, 95% CI: 1.53–2.41, p < 0.001) exhibiting significantly elevated anxiety levels. No statistically significant associations of pregnancy-related anxiety were found for educational level and occupation in this study cohort. The findings of this study highlighted the importance of psychosocial support and counseling, especially for older mothers and those with past obstetric complications, to reduce anxiety in an antenatal setting.

**Table 5:** Logistic regression analysis of pregnancy-related anxiety in Saudi Arabia.

Predictor variable	Odds ratio (OR)	95% CI	P-value
Age Group (Ref: 18–28 years)			
29–39 years	1.15	0.92 - 1.44	0.22
40–50 years	1.32	1.01 - 1.72	0.04*
51–60 years	1.08	0.75 - 1.56	0.68
Educational Level (Ref: Secondary)			
Higher education	0.89	0.72 - 1.10	0.28
Intermediate/Primary	1.12	0.85 - 1.47	0.41
Occupation (Ref: Housewife)			
Employed	1.05	0.84 - 1.31	0.67
Student	0.95	0.73 - 1.23	0.69
Unemployed	1.21	0.94 - 1.56	0.14
Previous Pregnancy Complications (Yes vs. No)	1.45	1.18 - 1.78	<0.001***
D&C Procedure (Yes vs. No)	1.30	1.05 - 1.61	0.02*
Anxiety About Delivery (Strongly Agree vs. Disagree)	2.10	1.65 - 2.67	<0.001***
Fear of Baby’s Health Issues (Strongly Agree vs. Disagree)	1.85	1.48 - 2.31	<0.001***
Fear of Pain During Delivery (Strongly Agree vs. Disagree)	1.92	1.53 - 2.41	<0.001***

**Figure 2:**
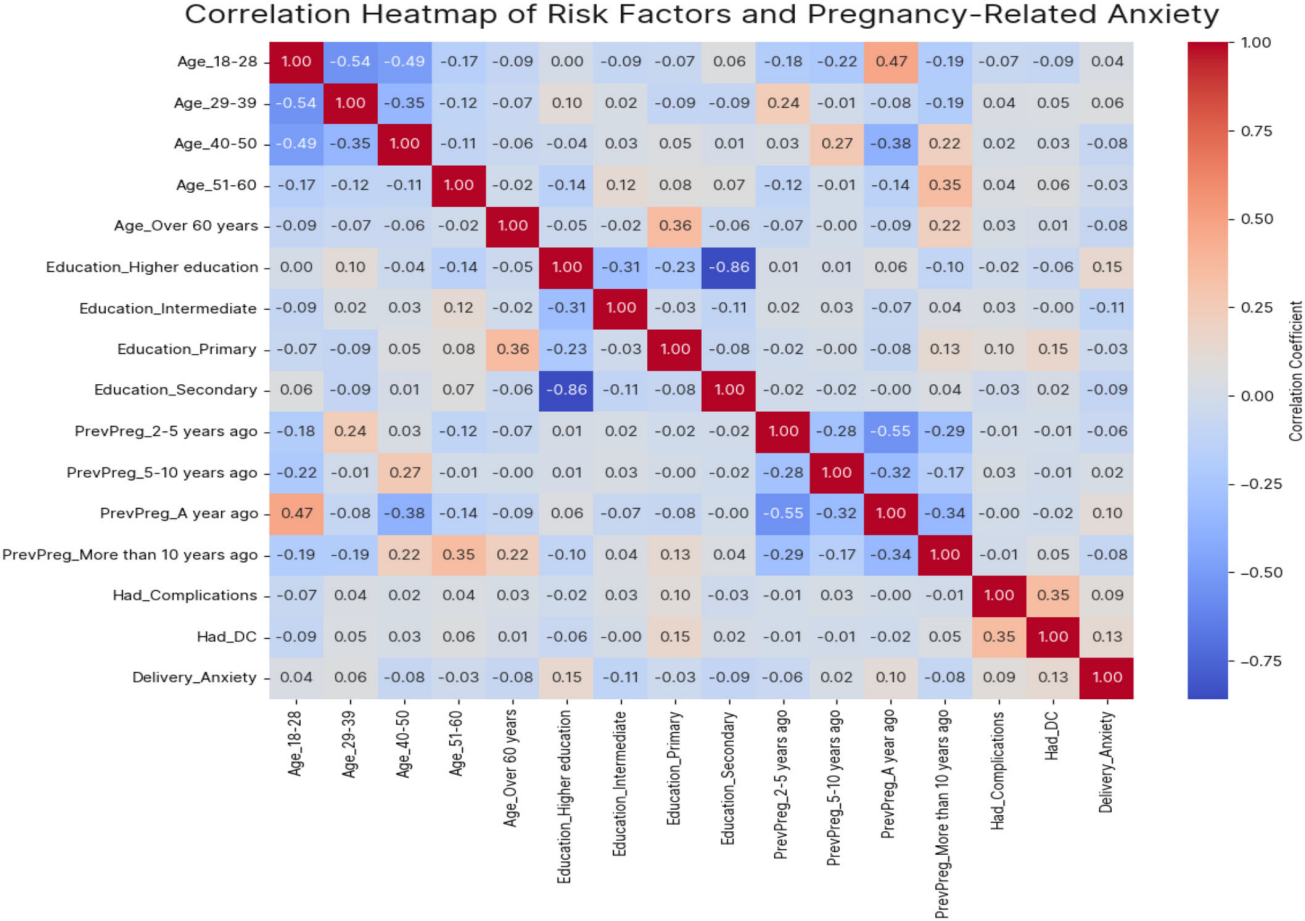
Correlation heatmap of the risk factors and PRA.

The correlation heatmap (**Figure 2**) revealed several key relationships between risk factors and pregnancy-related anxiety. The strongest positive correlations emerged for D&C procedures (r≈0.20-0.25) and pregnancy complications, reinforcing their status as significant anxiety predictors identified in our logistic regression analysis. Age exhibited a nuanced pattern among demographic factors: the 29–39 year group showed slight positive correlations with anxiety, while the over-60 cohort demonstrated negative correlations. Educational attainment displayed weak associations, with higher education showing a marginal positive correlation and intermediate education a negative correlation with anxiety levels. Notably, the timing of previous pregnancies showed differential associations - recent pregnancies (within 1 year) and those occurring 5–10 years prior correlated positively with anxiety, while pregnancies 2–5 years or >10 years prior showed slight negative correlations. The inter-variable correlations revealed expected strong negative associations between mutually exclusive categories (e.g., age groups and education levels), confirming appropriate variable coding. These findings suggest that while medical factors like D&C procedures and pregnancy complications demonstrate the most substantial linear relationships with anxiety, demographic characteristics appear to exert more subtle influences. The generally modest correlation magnitudes (all r<0.3) indicate that pregnancy-related anxiety likely arises from complex interactions between biological, psychological, and social determinants beyond those captured in our analysis. This underscores the need for comprehensive, multifactorial assessment in clinical settings when evaluating anxiety risk during pregnancy.

## 4 Discussion

The purpose of this national cross-sectional study is to identify the predictors of pregnancy-related anxiety in women in Saudi Arabia. Conclusions indicate an adequate burden of PRA; 71% of the participants reported significant concern related to delivery. The analysis identified several major predictors, indicating that older maternal age (40–50 years), a history of maternity complications, and pre-D&C processes increased the obstacles to PRA. In addition, specific apprehensions - concern about the distribution process, child’s health, and labor pain - primarily known as powerful psychological prophets, are known as more than doubling the possibility of experiencing significant anxiety. A notable secondary discovery was a significant decrease in the level of anxiety as pregnancy progressed in the weeks after pregnancy. These results collectively highlight that PRA is a prevalent maternal health issue in Saudi Arabia, shaped by a complex interplay of demographic, obstetric, and psychological factors.

PRA is highly prevalent, especially in developing countries (17), compared to generalized anxiety disorders; PRA is limited to the prenatal period and can affect maternal and fetal outcomes significantly (18). Tackling PRA is, therefore, critical in the Saudi Arabian context because of its immediate implications for the health of all pregnant women and potentially critical long-term effects on the health of mothers and children. This study aimed to determine PRA’s prevalence and risk factors in Saudi Arabia.

The findings revealed a significant proportion of participants (71.0%) experienced anxiety related to childbirth, with 39.3% strongly agreeing and 31.7% agreeing. It is well established that the relationship between obstetric complications and psychological distress during pregnancy is bidirectional and not unidirectional. However, our study found that a significant number of respondents,109(15.9%), had previous pregnancy complications, including gestational diabetes, hypertension, and postpartum hemorrhage, and these were significantly associated with increased pregnancy anxiety. The importance of these findings is heightened in the context of the healthcare system in Saudi Arabia, which, by addressing such complications, could contribute to improved maternal and fetal outcomes. Research indicates, for example, that higher resilience is linked to lower complication rates in women and better pregnancy outcomes (19) (Khalil et al.), similarly, a study by Hamzehgardeshi et al. revealed that during the COVID-19 pandemic, 21% of pregnant women suffered PRA, identifying factors such as the number of pregnancies, anxiety, grief, and social support as significant predictors of PRA (20).

Concerns regarding labor pain and contractions were even higher at 76.9%, with 42.4% strongly agreeing and 34.5% agreeing. Additionally, 59.7% of participants expressed anxiety about their body image post-delivery, reflecting a significant psychological burden. This increased anxiety over labor pain is in line with the results of previous studies, where fear of pain is considered one of the most frequent stressors among pregnant women, especially first-time mothers and those with the last traumatic birth experience (21, 22). Research has shown that the anticipation of labor pain was strongly associated with psychological and physiological responses, including elevated stress levels (21). Bagade et al. reported that 75% of pregnant women suffered from pregnancy-related anxiety, with worries about delivery, having disabled children, and postpartum appearance as significant sources of anxiety (22).

Concerns regarding their child’s health and appearance, mental health problems, physical defects, stillbirth, and loss of control during labor were also significantly (P < 0.05) expressed by participants. These underscore the importance of education and advocacy for expectant mothers.” Ibrahim et al. reported a higher fear of childbirth (FOC) among nulliparous women (80%) than multiparous women (67.8%). FOC showed a negative correlation with family support and routine prenatal care, highlighting the necessity of additional attention for high-risk women (23). In another study conducted by Abegaz et al., pregnancy-related anxiety was 43.9%, and it was significantly associated with low social support, primigravida, and intimate partner violence (24). The study identified gender education, violence against women, and social support as issues that needed to be addressed.

Another critical point is the gestational age distribution among participants, where 31.4% were at 39–40 weeks. The study demonstrated decreased levels of anxiety over the three trimesters with concerns regarding delivery from 47.7% at 35–36 weeks to 31.8% at 37–38 weeks (p=0.043). This trend implies that the further into pregnancy, the better-prepared women may feel for giving birth, possibly due to heightened interactions with healthcare or support groups. Scope of the study aligned with Haile et al. (25) and at an early stage, reducing anxiety emphasized the importance of early detection and treatment and collaboration with organizations. Wegbom et al. reported a significant effect of age, religion, trimester, and abortions/miscarriages (previous) on stress, anxiety, and depressive state of pregnant women. These highlighted the urgent need for mental health policies to alleviate anxiety among pregnant women (26).

**Figure 3:**
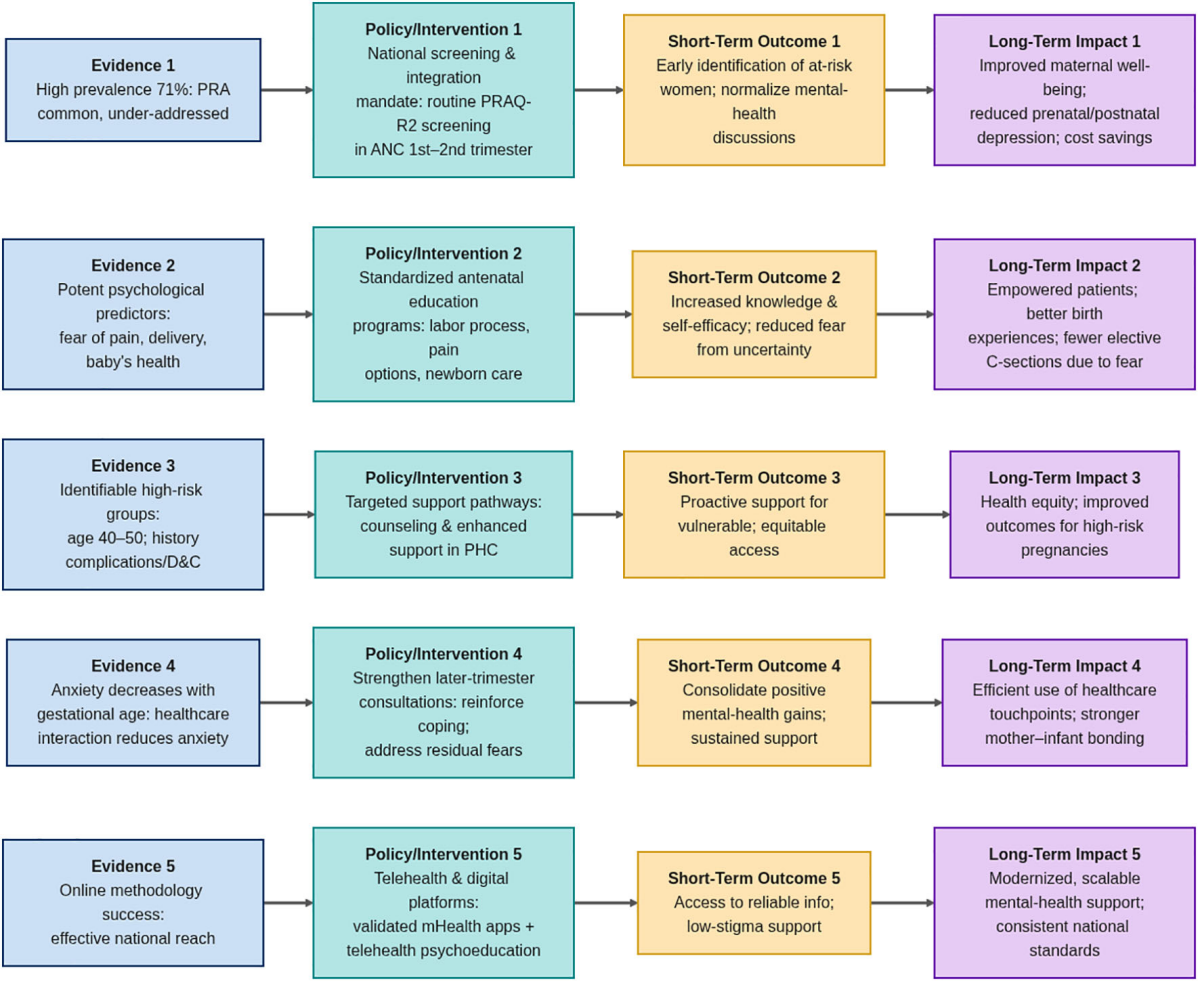
Conceptual framework: from evidence to action for reducing pregnancy-related anxiety in Saudi Arabia.

Based on the findings of the study, we propose ‘from evidence to action’ from ideological structure. This structure presented here provides a vital translation of our empirical findings in a strategic roadmap for tangible policy and healthcare reform (**Figure 3**). Our study anxiety is not a statistical discovery that affects participants-71% participants in a statistical study. The high prevalence of concerned anxiety is not a statistical discovery, but an immediate indicator of an important public health difference in the current prenatal care model. Framework describes this high proliferation in regular postpartum care as a clear call for immediate and systematic integration of mental health screening, which is cautious only on physical health beyond traditional.

In addition, the framework explains the identity of specific, robust predictions - such as pain, delivery, and fear of concerns for the child’s health - disclosing a primary driver of PRA: lack of uncertainty and preparation. This interpretation marks a significant shift from a reactive, treatment-based model to an active, educational, and empowerment-based strategy. By recommending standardized antenatal education, the framework posits that equipping women with knowledge and realistic expectations is a fundamental preventative measure, signifying a progressive approach in healthcare.

The interpretation of our data on high-risk groups (older mothers, those with obstetric complications) through this framework highlights an issue of health equity. This argues that a one-size-fits-all approach is inadequate and resources should be strategically targeted to ensure that the weaker population obtains sensitive, enhanced support, emphasizing the importance of personalized care.

Finally, lack of anxiety with pregnancy age is constantly interpreted as evidence of the medical value of healthcare contact. This confirmed the framework’s suggestion to improve quarterly consultations, indicating that the healthcare system itself is a strong place for interventions that can be easily changed. In short, this structure explains our scientific results not as isolated facts, but as a more comprehensive, preventive, and interconnected framework for creating a more supportive, preventive, and justified maternal mental health system in Saudi Arabia. The final explanation is that reducing the PRA is achieved through evidence-informed structural changes in prenatal care distribution.

The high prevalence of pregnancy-related anxiety (71%) must be contextualized. While elevated, it aligns with regional trends and may reflect methodological influences, including measurement sensitivity and sampling bias. Crucially, a comprehensive understanding requires moving beyond individual risk factors. A multi-level public health perspective is essential, one that considers the roles of health system structures (e.g., integrated mental health care), social support networks, and broader societal and gender norms that shape maternal wellbeing. Effective intervention must therefore target these systemic and social determinants alongside individual support.

Our findings call for concrete, system-integrated actions within the Saudi maternal healthcare framework. We recommend: (1) the mandatory inclusion of brief, validated anxiety screening into national antenatal care guidelines, with clear referral pathways; (2) the development of standardized, culturally-grounded education modules addressing specific high-prevalence fears, delivered via primary care centers and digital platforms like the *Sehaty* app; (3) the establishment of targeted support protocols for high-risk groups, such as older primigravidas and women with prior complications; and (4) investment in training programs to build frontline providers’ competency in recognizing and providing first-line psychological support. By translating evidence into these specific, actionable recommendations, we provide a roadmap for systematically strengthening maternal mental health care in Saudi Arabia.

### 4.1 Limitations

This study has a number of key limitations. First, as a cross-sectional study, we could not infer causality from the identified factors to pregnancy-related anxiety. Second, population-level weighted national samples like this are generally skewed in terms of age and education distribution because the convenience of an Internet-based recruitment strategy bias selection towards younger, more educated, urban women with experience using digital technology at the expense of older, less-educated rural populations who may have less access to information via digital means. This is a limitation to the external validity of our results, and accordingly, the prevalence and profile of predictors we reported cannot be simply generalized to Saudi pregnant women. On an equity perspective, this sampling bias is of great concern as it may systematically exclude populations who are already encountering obstacles to health and psychosocial supports and underestimate the true burden and specific determinants of anxiety in these most vulnerable populations. Third, a self-administered questionnaire might be subject to social desirability bias. Fourth, we used participants’ recall of obstetric history which may cause recall bias. Lastly, while the study found specific predictors of interest, it’s possible that confounding factors not measured in this sample (e.g., nuanced social support indicators, family-hold income levels and partner relationship quality) exert effects over the observed associations. However, the study offers important national coverage and indicates a requirement for further inclusive and community-engaged research to fully comprehend and respond to PRAS across all sectors of Saudi society.

## 5 Conclusion

This national study confirms that pregnancy-related anxiety is widespread in women in Saudi Arabia. There is a critical concern focused on the child’s birth, labor pain, and postpartum body image. Identification of important predictions provides the necessary evidence for targeted treatments.

These findings require changes in the Saudi healthcare system, ranging from awareness to actionable strategies. Regular postpartum care is needed to integrate regular PRA screening, implement standardized educational programs to address specific fears, and set up support routes dedicated to high-risk women. By translating these evidence-based insights into clinical practice and policy, the healthcare provider can significantly reduce maternal anxiety, which can improve both maternal welfare and newborn outcomes. This study not only depicts the burden of PRA but also provides a clear framework for the creation of a more supportive and active maternal health infrastructure in Saudi Arabia.

## Data Availability

The raw data supporting the conclusions of this article will be made available by the authors, without undue reservation.
