# Supplementary material for: Pregnancy-related anxiety in Saudi women: a national study of prevalence, predictors, and a framework for action
Source: Front Psychiatry. 2026 Feb 26;17:1716720. doi: 10.3389/fpsyt.2026.1716720 (PMC12979941; doi:10.3389/fpsyt.2026.1716720)
Supplement: Supplementary file 1 [file SupplementaryFile1.docx]

**Pregnancy-Related Anxiety in Saudi Women: A National Study of Prevalence, Predictors, and a Framework for Action**

# **Questionnaire**

| Hello,  We are from all over Saudi Arabia. We would like to conduct a study to estimate the prevalence of pregnancy-related anxiety among pregnant women in Saudi Arabia. In case you would proceed to answer this questionnaire, your personal information is preserved and will not be used for any purpose other than research. You have the right to accept or reject participation in this questionnaire.  If you live in Saudi Arabia and your age is 18 y/o or above, please fill out the questionnaire if you agree to participate:   - Agree - Disagree |
| --- |
| 1. **Sociodemographic data** |
| 1. Age  - 18-28 - 29-39 - 40-50 - 51-60 - Over 60 years |
| 1. Gender  - Male - Female |
| 1. Educational level  - Primary - Intermediate - Secondary - Higher education |
| 1. Occupation  - Student - Housewife - Retired - Employed - Health worker - Unemployed |
| 1. **Obstetric History and past history of anxiety** |
| 1. Date of previous pregnancy?  - A year ago - 2-5 years ago - 5-10 years ago - More than 10 years ago |
| 1. Length of pregnancy?  - 35-36 weeks - 37-38 weeks - 39-40 weeks - 41-42 weeks - 43 weeks |
| 1. Have you ever undergone a procedure called Dilatation and Curettage (D&C)?  - Yes - No |
| 1. Were there any complications during the previous pregnancy?  - Yes - No |
| 1. If yes, please specify……………. |
| 1. **Pregnancy Anxiety Questionnaire- Revised-2** |
| 1. I am anxious about the delivery.  - Strongly agree - Agree - Neutral - Disagree - Strongly disagree |
| 1. I am worried about the pain the of contractions and the pain during delivery.  - Strongly agree - Agree - Neutral - Disagree - Strongly disagree |
| 1. I am worried about the fact that I shall not regain my figure after delivery.  - Strongly agree - Agree - Neutral - Disagree - Strongly disagree |
| 1. I sometimes think that our child will be in poor health or will be prone to illnesses.  - Strongly agree - Agree - Neutral - Disagree - Strongly disagree |
| 1. I am concerned about my unattractive appearance.  - Strongly agree - Agree - Neutral - Disagree - Strongly disagree |
| 1. I am worried about not being able to control myself during labour and fear that I will scream.  - Strongly agree - Agree - Neutral - Disagree - Strongly disagree |
| 1. I am anxious about the delivery because I have never experienced one before.  - Strongly agree - Agree - Neutral - Disagree - Strongly disagree |
| 1. I am afraid the baby will be mentally handicapped or will suffer from brain damage.  - Yes - No - Maybe |
| 1. I am afraid our baby will be stillborn or will die during or immediately after delivery.  - Strongly agree - Agree - Neutral - Disagree - Strongly disagree |
| 1. I am afraid that our baby will suffer from a physical defect or worry that something will be physically wrong with the baby.  - Yes - No |
